# Ion beam etching redeposition for 3D multimaterial nanostructure manufacturing

**DOI:** 10.1038/s41378-019-0052-7

**Published:** 2019-04-22

**Authors:** B. X. E. Desbiolles, A. Bertsch, P. Renaud

**Affiliations:** Laboratory of Microsystems LMIS4, Ecole Polytechnique Fédérale de Lausanne (EPFL), Lausanne, Switzerland

**Keywords:** Nanofabrication, Ion beam etching, Redeposition, Multimaterial, Multilayer, Nanoscale devices, Nanoscale materials

## Abstract

A novel fabrication method based on the local sputtering of photoresist sidewalls during ion beam etching is presented. This method allows for the manufacture of three-dimensional multimaterial nanostructures at the wafer scale in only four process steps. Features of various shapes and profiles can be fabricated at sub-100-nm dimensions with unprecedented freedom in material choice. Complex nanostructures such as nanochannels, multimaterial nanowalls, and suspended networks were successfully fabricated using only standard microprocessing tools. This provides an alternative to traditional nanofabrication techniques, as well as new opportunities for biosensing, nanofluidics, nanophotonics, and nanoelectronics.

## Nanofabrication: 3D multimaterial nanostructures manufactured by ion beam etching redeposition

The local sputtering of photoresist sidewalls during ion beam etching enables nanoscale structures to be fabricated. Top-down manufacturing is a scalable approach for the synthesis of nanoscale structures with a range of promising functional properties, and there is an ever growing need to expand the diversity of structures that can be achieved. A team from Ecole Polytechnique Fédérale de Lausanne led by Philippe Renaud now demonstrates how sidewall photoresists might be used to grow structures from a substrate. During ion beam etching of a silicon substrate, the etched material is partly redeposited on the photoresist sidewall. By stripping out the photoresist, sub-100 nm features are achieved, including those composed of multiple materials via the deposition of different materials on the substrate prior to etching.

## Introduction

Recent advances in nanofabrication have led to significant discoveries in a wide range of areas, such as biosensing, nanofluidics, nanophotonics, and nanoelectronics^1^. Most of the nanofabrication techniques used today can be classified in two main categories: bottom-up and top-down^2^. Bottom-up fabrication methods use the interactions between molecules or atoms to build-up complex nanoscale assemblies in two or three dimensions. Atomic layer deposition^3^, molecular self-assembly^4^, and DNA self-assembly^5^ are common examples of bottom-up nanofabrication methods. These processes can cover large areas with nanoscale features but suffer from a lack of control, particularly in the geometry of assembled structures.

Top-down approaches are based on the structuration of materials at the nanoscale, starting from a bulk material that was shaped by a series of steps that often include lithography, dry or wet etching, oxidation, and metallization, among others. High-resolution lithography methods are often used, such as optical lithography^6^, e-beam lithography^7^, soft lithography^8^, nanoimprint lithography^9^, and scanning probe lithography^10^. Such methods allow for control over the dimensions, shapes, and characteristics of nanostructures over a length scale four orders of magnitude larger than an individual structure^11^. However, limitations include high operating costs, low scalability, and lack of versatility—especially for three-dimensional (3D) nanostructure manufacturing.

Using top-down microfabrication technologies in an “unconventional” but ingenious way allows for the manufacture of sophisticated nanostructures without pushing techniques to the limit or using complex and expensive dedicated manufacturing processes. For example, single-material sub-micron two-dimensional (2D) structures were successfully manufactured by evaporating a thin film onto a substrate covered with self-assembled monolayers of polystyrene microspheres^12^. The microspheres served as a mask for the pattern to be created in the interstitial spaces during the deposition step. The resulting 2D triangular structures were 500 nm in size and covered a large area of the substrate. In order to gain more control over structural shape and decrease the critical dimension, openings were patterned on a suspended gold (Au) membrane. Through tilted-angle Au evaporation, 100 nm single-material features of various geometries were obtained on a large-scale and at a low operating cost^13^. Even sub-10-nm nanostructures composed of several materials were fabricated using ingenuities. Chemical-mechanical polishing performed on tapered microstructures covered with a stack of several materials led to 5-nm-thick concentric Ti-Au-Ti nanorings at the wafer scale^14^. Similar examples can be found in the literature; however, none describe a reliable method to produce complex 3D multimaterial nanostructures at the wafer scale.

In this work, the use of local sputter-redeposition on photoresist sidewalls during ion beam etching^15–17^ is proposed to manufacture multimaterial 3D nanostructures. Using this method, nanostructures of various shapes, profiles, heights, thicknesses, and complexity can easily be fabricated at the wafer scale in a short period of time. The simplicity of this process, using standard microprocessing tools only, makes the nanofabrication of complex structures fast and accessible.

The potential of the present method was first investigated for silicon (Si) nanostructures. The fabrication of multilayered structures composed of nonsilicon materials—hardly achievable using standard nanofabrication processes—is discussed within. The method was also used to manufacture complex structures optimized for specific applications.

## Results

### Single-material nanostructures

**Fig. 1:**
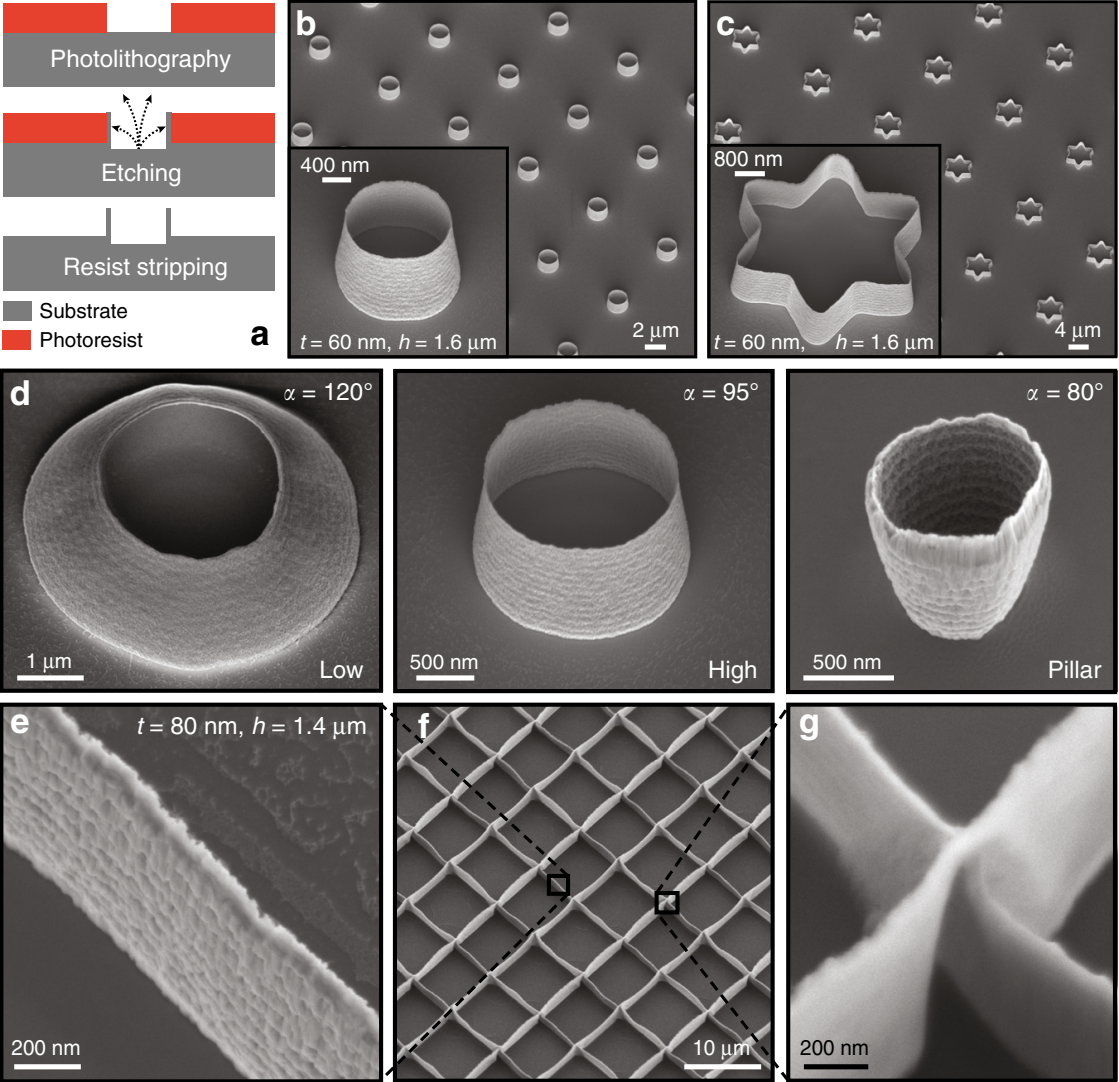
Structures created by Si redeposition on photoresist sidewalls during Ar^+^ ion beam etching. **a** Illustration of the process flow used to create Si nanostructures: during ion beam etching, the photoresist sidewalls are sputtered by the etched material and after resist stripping, nanostructures are obtained. SEM images of **b** cylinder arrays, **c** star arrays composed of 60-nm-thick and 1.6-μm-high walls, and **d** profiles obtained by changing the dose from low to high during photolithography. The structure profile can even become negative by performing the redeposition step on a pillar rather than a resist opening. The angle *α* is defined between the substrate and the cylinder wall. For a vertical wall *α* = 90°. **e**−**g** SEM images of 80-nm-thick and 1.4-μm-high intersecting nanowall arrays. Tilt angle of 30°. SEM scanning electron microscope

Figure 1a illustrates the fabrication method studied in this work. A photoresist layer was patterned by standard photolithography onto an Si substrate, which was then bombarded with argon ions (Ar^+^) during ion beam etching. Not only did this step lead to the etching of uncovered areas, but it also induced the local redeposition of etched materials onto the photoresist sidewalls. This material redeposition actually created the 3D nanostructures, and a final photoresist stripping completed the fabrication process.

Figure 1b is a scanning electron microscope (SEM) image of tapered cylinders composed of 60-nm-thick and 1.6-μm-high Si walls arranged in an array. These structures were created by patterning 1.5 μm circular openings in a photoresist layer prior to ion beam etching. The openings can be various shapes, as illustrated in Fig. 1c and Fig. S1. The dimensions achieved for the Si cylinders in this study are summarized in Table 1. Walls as thin as 6 nm and as thick as 200 nm were successfully created with heights varying from 200 nm to 15 μm and diameters varying from 300 nm to 200 μm.

**Table 1:** Summary of successfully manufactured Si cylinder dimensions

	Wall thickness [nm]	Diameter [nm]	Height [nm]
Min	6	300	200
Max	200	200,000	15,000

The profile of redeposited structures can also be tuned by adjusting the dose of the photolithography step. A high dose leads to straight walls, whereas a low dose induces curved walls, as shown in Fig. 1d. By patterning pillars instead of holes, the structure profile can even be inverted (cf. Fig. 1d). Note that the nanostructure surfaces are the exact replica of the photoresist sidewalls. As demonstrated in Figure S2, their surface roughness can be smoothened by reflowing the photoresist before redeposition. It is not an intrinsic limitation of the fabrication process.

As shown in Fig. 1e–g, 80-nm-thick intersecting nanowall networks were obtained by defining photoresist lines during photolithography and repeating the presented method twice. The fabrication process was simple, scalable, and led to homogeneous structures at the wafer scale.

### Multimaterial nanostructures

**Fig. 2:**
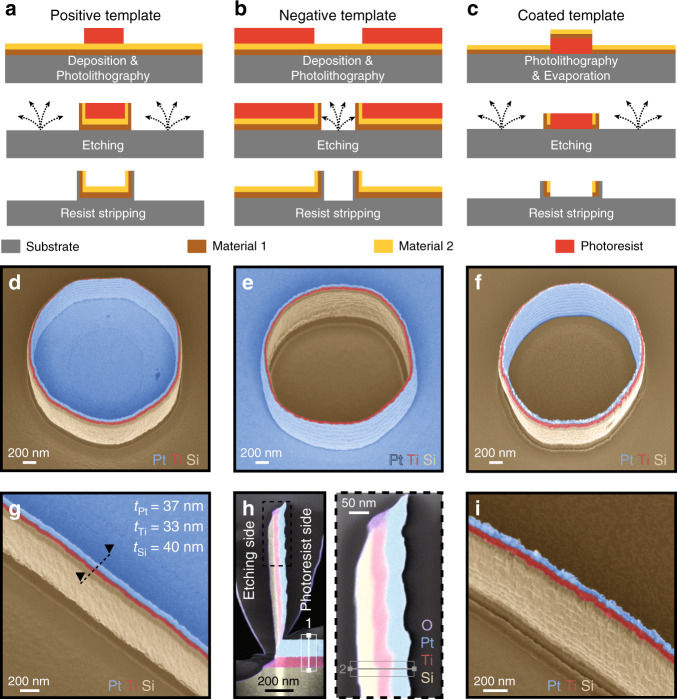
Multilayered structures created by the redeposition of different materials. **a**−**c** illustrate three different process flows and **d**−**f** show the respective structures obtained by these three methods, each differing by the location of the different materials used in the final structures. More precisely, **a** layers of different materials were deposited before patterning pillars by photolithography; **b** layers of different materials were deposited before patterning openings by photolithography; **c** layers of different materials were deposited after patterning pillars by photolithography. **g** Nanowall created using layers of Si, Ti, and Pt corresponding to the fabrication process presented in (**a**); **h** TEM image combined with an EDX spectroscopy map of a redeposited nanowall cross-section located with dashed lines in (**g**). The photoresist was on the right side of the nanowall during ion beam etching. A carbon layer was locally deposited onto the wall to protect the structure during sample preparation; **i** nanowall created using layers of Si, Ti, and Pt corresponding to the fabrication process presented in (**c**). Tilt angle of 30°. The colors in images (**d**)−(**g**) and (**i**) were added in postprocessing based on the contrast resulting from the detection of back-scattered electrons. In **(h**), the colors directly result from EDX analysis. TEM transmission electron microscopy, EDX energy dispersive X-ray spectroscopy

Multimaterial nanostructures can be produced by combining the studied fabrication method with standard deposition processes, as illustrated in Fig. 2a–c. In Fig. 2a, several layers of different materials were deposited onto the substrate prior to patterning the photoresist pillars. During ion beam etching, the material of the top layer was first redeposited on the photoresist sidewalls, followed by the materials in deeper layers. The substrate material was redeposited last.

As an example, an SEM image is presented in Fig. 2d showing a cylindrical wall obtained using this method on a 2 μm diameter photoresist template pillar patterned on top of layers of titanium (Ti) and platinum (Pt) deposited on an Si substrate. The interior of the obtained cylindrical structure was composed of Pt, while the outside was composed of Si and delimited by a sharp multimaterial nanowall composed of Si-Ti-Pt layers. In Fig. 2b, e, openings in the photoresist were patterned, rather than pillars, using the same sequence of deposited metal layers on an Si substrate (as in Fig. 2d). The obtained redeposited structures have similar shapes and dimensions to the one presented in Fig. 2d, though the order of the materials in this structure were opposite, with the inner layer of the obtained structure being Si and the outer layer being Pt. The Ti layer remained in the middle of the sharp wall. Similarly, Fig. 2c, f demonstrates what occurs when the metal layers are deposited after the photolithography step. Multimaterial cylinders composed of up to six different layers were successfully fabricated, as shown in Figure S4-A.

If lines are patterned by photolithography, centimeter-long multimaterial nanowalls can be made, as shown in Fig. 2g, i.

A cross-section of a redeposited multilayered structure was characterized by transmission electron microscopy (TEM) combined with energy dispersive X-ray spectroscopy (EDX). Figure 2h shows how the layers of different materials deposited on the substrate were redeposited on photoresist walls during ion beam etching, with the topmost layer undergoing etching and redeposition first. The redeposition was uniform along the wall and became thinner at the intersection with the planar layers. Native titanium oxide was observed at the end of the Ti layer in contact with air. The detailed composition of each redeposited layer obtained by quantitative EDX analysis is summarized in Table S1.

Performing EDX line scan analysis on three different samples at the two positions shown in Fig. 2h allowed for the accurate measurement of the thickness of each redeposited layer. Table 2 presents the redeposition ratio and rate obtained by measuring the thickness of the initial and redeposited layers for each material. Materials that were difficult to etch have slow redeposition rates and high redeposition ratios.

**Table 2:** Summary of the redeposition ratios and rates for Ti, Pt, and Si during ion beam etching using Ar^+^ ions

Material	Thickness [nm]		Redeposition ratio [%]	Etching rate [nm/min]	Redeposition rate [nm/min]
	On substrate	On wall			
Ti	80	33.3	41.6	35.5	14.8
Pt	115	36.6	31.8	92	29.3
Si	110	40	36.3	73.3	26.7

Fast Fourier transform pattern analysis of high-resolution TEM images demonstrated that the materials composing the walls of the nanostructure were highly amorphous, while those constituting the substrate were more crystalline (cf. Figure S3). The ion beam etching step induced a change of crystallinity in the redeposited materials.

### Complex nanostructures

**Fig. 3:**
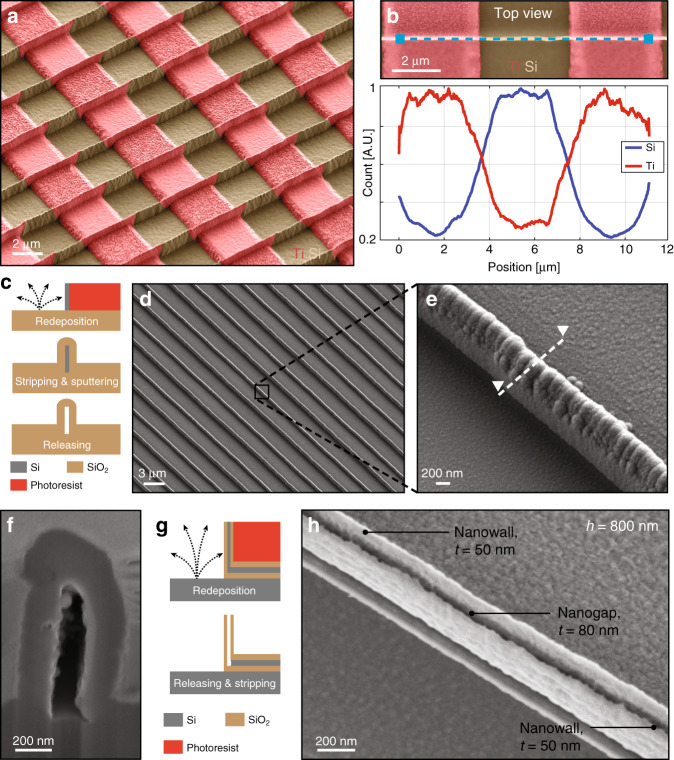
Complex nanostructures manufactured by the redeposition of several materials. **a** SEM image of nanowalls alternately composed of Si and Ti along the longitudinal direction. Perpendicular to the walls, prepatterned Ti lines on the Si substrate were used to build the multimaterial walls during ion beam etching. **b** EDX line scan analysis of the nanowall material composition. The blue dashed line in the SEM image indicates the location of the analysis. **c** Illustration of the fabrication process used to manufacture SiO_2_ nanochannels. An SiO_2_ layer was sputtered on an Si sacrificial template made by ion beam etching redeposition. The template was then removed by isotropic Si dry etching. **d**, **e** SEM images of the resulting SiO_2_ nanochannels. **f** SEM image of the nanochannel cross-section indicated by the dashed line in (**e**) after Si release. **g** Illustration of the fabrication process used to manufacture two SiO_2_ nanowalls with a nanogap in between. An Si sacrificial template was fabricated in the middle of two SiO_2_ nanowalls by ion beam etching redeposition. After release, the nanogap was obtained. **h** SEM image of two 50-nm-thick and 800-nm-high SiO_2_ nanowalls separated by an 80 nm gap. Tilt angle of 30°. The colors have been added by postprocessing based on the contrast resulting from the detection of back-scattered electrons. SEM scanning electron microscope, EDX energy dispersive X-ray spectroscopy

Combined with conventional micro electromechanical systems fabrication techniques, this simple fabrication method can lead to the manufacture of complex 3D multimaterial nanostructures. The SEM image presented in Fig. 3a shows nanowalls alternately composed of Ti and Si along the longitudinal direction. This was performed by patterning the substrate with Ti lines prior to the fabrication of the nanostructures by redeposition and defining line openings perpendicular to the Ti lines by photolithography. During ion beam etching, materials from the Ti lines and Si substrate were locally redeposited on the photoresist sidewalls, leading to the formation of nanowalls made alternately of Ti and Si along the longitudinal direction. As Ti was not etched entirely during ion beam etching, Ti lines could still be observed on the substrate. The alternating Ti−Si composition of the nanowalls along the longitudinal direction has been validated by EDX line scan analysis, as shown in Fig. 3b.

Nanochannels of any length and shape can be obtained by covering Si nanowalls with a silicon dioxide (SiO_2_) layer and subsequently selectively etching the Si nanostructures (cf. Fig. 3c). Such nanochannels can be made at the wafer scale, as illustrated in Fig. 3d, e. A cross-section of this type of structure was obtained by focused ion beam (see Fig. 3f), which highlighted a sub-200 nm cavity trapped between SiO_2_ walls.

Figure 3h presents an SEM image of two 50-nm-thick and 800-nm-high SiO_2_ walls separated by an 80 nm gap. This was performed by depositing an Si layer sandwiched between two SiO_2_ layers on the substrate before photolithography. After the redeposition, occurring during the Ar^+^ ion beam etching process, a multilayer nanowall composed of SiO_2_-Si-SiO_2_ was obtained. The Si layer was sacrificial and was etched away to create the nanogap separating the two nanowalls (cf. Fig. 3g).

Repeating such a process twice can lead to multimaterial suspended structures. In Figure S4-B, suspended Ti nanowalls alternately joined by a thin membrane can be observed on Si nanowall networks.

## Discussion and applications

Using a simple fabrication method based on the redeposition of materials during Ar^+^ ion beam etching, 3D nanostructures can be manufactured at the wafer scale in a very short amount of time. The shape, dimension, and profile can be engineered to specific applications without using nanolithography techniques, but rather using standard microprocessing tools only, resulting in a decreased cost compared to the use of technologies such as e-beam. Various materials, from metals to ceramics, can be patterned into 3D sub-100 nm nanostructures. Pt, Ti, Si, SiO_2_, Au, IrOx, and Al have been successfully used in the redeposition step. This list can certainly be extended to any sputterable material, resulting in unprecedented freedom in the choice of nanostructure fabrication materials. This diversity brings significant improvements to the current state of the art for nanostructure manufacturing, where materials are limited for many nanopatterning technologies. Additionally, the nanofabrication method described here can be used advantageously to replace more complex manufacturing processes previously presented in the literature.

### Biosensing

For instance, gold nanostraws with a geometry similar to the structures presented in Fig. 1b were used as plasmonic structures to optoporate cell membranes and provide intracellular access^18^. The nanostraws were fabricated using secondary electron lithography generated by ion beam milling, a top-down technique allowing precise control over the geometry of the hollow structure, though this process was hardly scalable. With some adjustments, the method presented here could be used to fabricate such gold nanocylinders at the wafer scale.

Not only does this fabrication method allow for the manufacture of 3D nanostructures composed of nonstandard materials, but it also enables the easy patterning of multimaterial 3D nanostructures, potentially increasing applications. With minor adjustments, the multimaterial walls presented in Fig. 3a could be used to fabricate 3D electrodes, thermal oscillators, or electrostatic actuators. Specifically, the structure shown in Fig. 2e is very similar to the multimaterial feature built by Van Dersarl and Renaud^14^, where “unconventional” top-down techniques were used to fabricate a 10-nm-thick protruding gold nanoring sandwiched between two Ti layers and one SiO_2_ layer surrounding a planar electrode. The purpose was to optimize the cell−electrode interface and improve the electrophysiological recording quality. This was done by performing chemical-mechanical polishing on a micro-patterned surface covered with Ti-Au-Ti-SiO_2_ layers. Even if the fabrication method used was ingenious, it was limited by the lack of homogeneity induced during the polishing step. In order to provide greater homogeneity to the structures over the entire wafer area, the redeposition process described here would be advantageous to fabricate similar structures using openings in photoresist layers as a template for multimetal redeposition.

This novel technology also provides new opportunities for the detection of electrochemically active molecules using nanoscale redox cycling structures, where two electrodes separated by a nanogap are usually used to collect the electrochemical current induced by redox reactions at the interface. Interestingly, the sensor sensitivity is improved by reducing the nanogap dimensions, due to a shorter diffusion time between the two electrodes^19^. In the literature, embedded nanochannels made by conventional nanofabrication techniques^20,21^, or protruding sensors produced by focused ion beams^19^ are used. However, the embedded geometry often prevents a fast and direct collection of biological molecules, whereas the fabrication of focused ions beam-based devices is not scalable. Following the fabrication process illustrated in Fig. 3g—with Pt-SiO_2_ stacks instead of SiO_2_—protruding and insulated vertical electrodes, separated by a nanogap could potentially be manufactured at the wafer scale by ion beam etching redeposition. These electrodes could easily be integrated with microfluidic chips and guarantee a minimal distance with the molecule of interest, enhancing the temporal resolution of the recording. Furthermore, it has been demonstrated that the smoothening of the photoresist sidewalls before ion beam etching leads to a more homogeneous redeposition with a smoother surface roughness. This could drastically decrease the minimal nanogap dimension achievable with this technology and significantly improve the sensor sensitivity.

### Nanofluidics

The selective removal of a specific layer in a multimaterial nanostructure allows for an increase in the degree of complexity of achievable features. As shown in Fig. 3c−f, nanofluidic channels can be fabricated over a large area by using redeposited silicon as a sacrificial layer. In the literature, similar nanochannels from 50 to 300 nm wide were obtained by reactive ion etching of a quartz substrate protected by a photoresist mask previously patterned using a stepper^22^.

### Nanophotonics

Manufactured in a similar manner, the structures in Fig. 3h are comparable to the vertically oriented plasmonic nanogaps fabricated by metal deposition and anisotropic etching used for surface-enhanced Raman spectroscopy^23^. The method presented here is an alternative to fabricating these types of nanopatterns in a short period of time without the intervention of expensive and time-consuming processes, making such devices more accessible.

By repeating the redeposition process, the obtained structures become even more sophisticated. Suspended multimaterial nanowall networks can be obtained quickly at the wafer scale. Such structures appear similar to the photonic components found in the literature. In particular, it was demonstrated that the direct patterning of titanium dioxide using femtosecond laser pulses led to comparable patterns^24^. Using the redeposition of the photoresist sidewalls during ion beam etching could potentially simplify the fabrication process and make it more scalable.

## Conclusion

In this work, a novel “unconventional” nanofabrication method exploiting the redeposition during Ar^+^ ion beam etching was proposed to manufacture 3D multimaterial sub-100 nm nanostructures at the wafer scale. This simple fabrication process provided a rapid and lower-cost alternative to conventional nanofabrication techniques, with outstanding freedom in the choice of materials. Complex nanostructures such as nanochannels, multimaterial nanowalls, as well as suspended multimaterial networks were successfully fabricated in a few steps using the presented method. The potential applications were discussed based on similar examples from the literature. This nanofabrication method being integrable within other process flows may allow for new technological developments in the field of nanotechnologies.

## Methods

### Fabrication of multilayered structures

A standard four-inch Si substrate (thickness 525 μm, orientation 〈100〉, p-doped) was sputter-coated by 100 nm of Ti and 100 nm of Pt using a Spider 600 sputter-coater (Pfeiffer Vacuum, France). The substrate was spin-coated with a 1.4-μm-thick layer of AZ nLof 2020 photoresist (MicroChemicals, Germany) using an EVG150 spin-coater/developer (EVG, Austria) and was exposed with an i-line VPG direct laser writer (Heidelberg, Germany) using doses between 50 and 150 mJ/cm^2^. After a post-exposure bake at 110 °C with a 50 μm proximity gap for 75 s, the wafer was developed over 46 s using an AZ 726 MIF commercial developer (MicroChemicals, Germany) and was dispensed using an EVG150 coater/developer. The sample was then bombarded with Ar^+^ ions at an incident angle of 0° using an IBE350 ion beam etcher (Veeco, USA). Materials from the substrate were etched and redeposited on the photoresist sidewalls. The photoresist was finally stripped using a 500 W O_2_ plasma (O_2_ flow 400 ml/min) for 7 min with a TePla 300 microwave plasma system (PVA TePla, Germany).

Structures higher than 3 μm were fabricated using a thick layer of AZ40XT photoresist (MicroChemicals, Germany).

### Microscopy

All SEM images were taken with a Merlin SEM (Zeiss, Germany). In order to highlight the difference in contrast between the two materials, secondary electrons and back-scattered electrons were collected during image acquisition. For the EDX analysis leading to Fig. 3b, the voltage used was 3 kV, which was sufficient to observe Lα Ti (0.452) and the Kα Si (1.739) X-ray characteristics.

Scanning transmission electron microscopy images and the corresponding EDX analysis (cf. Fig. 2h) were done using a Tecnai Osiris TEM (FEI, USA). In order to obtain reliable map data, the acquisition time was set to 10 min/image during EDX analysis.

## Supplementary information

Supplementary information

Figure S1

Figure S2

Figure S3

Figure S4

Table S1
