# Supplementary material for: Ion beam etching redeposition for 3D multimaterial nanostructure manufacturing
Source: Microsyst Nanoeng. 2019 Apr 22;5:11. doi: 10.1038/s41378-019-0052-7 (PMC6475643; doi:10.1038/s41378-019-0052-7)
Supplement: Supplementary file 1 — Supplementary information [file 41378_2019_52_MOESM1_ESM.docx]

**Supplementary information**

Ion beam etching redeposition for 3D multimaterial nanostructure manufacturing

B.X.E. Desbiolles^[[1]](#footnote-1)^, A. Bertsch1, and P.Renaud1

# Single material nanostructures

**Figure S1:** *Structures made by Si redeposition on photoresist sidewalls during* Ar^+^ *ion-beam etching.* SEM images of **A)** square arrays, **B)** cross arrays, **C)** star arrays, and **D)** cylinder arrays composed of 60nm thick walls. *Tilt angle of 30°.*

**Figure S2:** *Smoothening of the nanostructure surface roughness by photoresist reflow (160°C for 2 minutes).* SEM images of the photoresist mask non-treated **A)**, or treated **B)**, by thermal reflow. **C)** and **D)** show typical Si nanostructures obtained respectively without, or with, thermal reflow. *Tilt angle of 30°.*

# Crystallinity and composition of the nanostructures

**Figure S3:** *Fast Fourier transform pattern analysis of high resolution TEM images.* **A)** Diffraction pattern of a Ti layer evaporated on the substrate before the redeposition process. **B)** Diffraction pattern of the same layer after the redeposition process. The Ti layer is more amorphous after ion-beam etching redeposition.

**Table S1:** Composition of the redeposited layers presented in Figure 2-H measured by EDX.

| **Layer** | **Si [%]** | **Ti [%]** | **Pt [%]** | **Ar [%]** | **Traces Cu, Cr, Fe [%]** |
| --- | --- | --- | --- | --- | --- |
| **Si** | 88.5 | 4.8 | 3.0 | 1.5 | 2.2 |
| **Ti** | 1.8 | 82.6 | 11.5 | 1.4 | 2.7 |
| **Pt** | 8.3 | 1.1 | 87.3 | 1.0 | 2.3 |

# Multimaterial complex nanostructures

**Figure S4:** *Multimaterial complex nanostructures made by redeposition on photoresist sidewalls during* Ar^+^ *ion-beam etching.* **A)** SEM image of a multilayered cylinder composed of five different materials: IrOx, Ti, Au, Al, and Si **B)** SEM image of suspended Ti nanowalls, alternately joined by a thin Ti membrane standing on a Si nanowall networks. The cross-sections 1 and 2 are illustrated in the insert. *Tilt angle of 30°.*

1. Laboratory of Microsystems LMIS4, Ecole Polytechnique Fédérale de Lausanne (EPFL), Lausanne, Switzerland. [↑](#footnote-ref-1)
