# Supplementary figures and images for: Ion beam etching redeposition for 3D multimaterial nanostructure manufacturing
Source: Microsyst Nanoeng. 2019 Apr 22;5:11. doi: 10.1038/s41378-019-0052-7 (PMC6475643; doi:10.1038/s41378-019-0052-7)

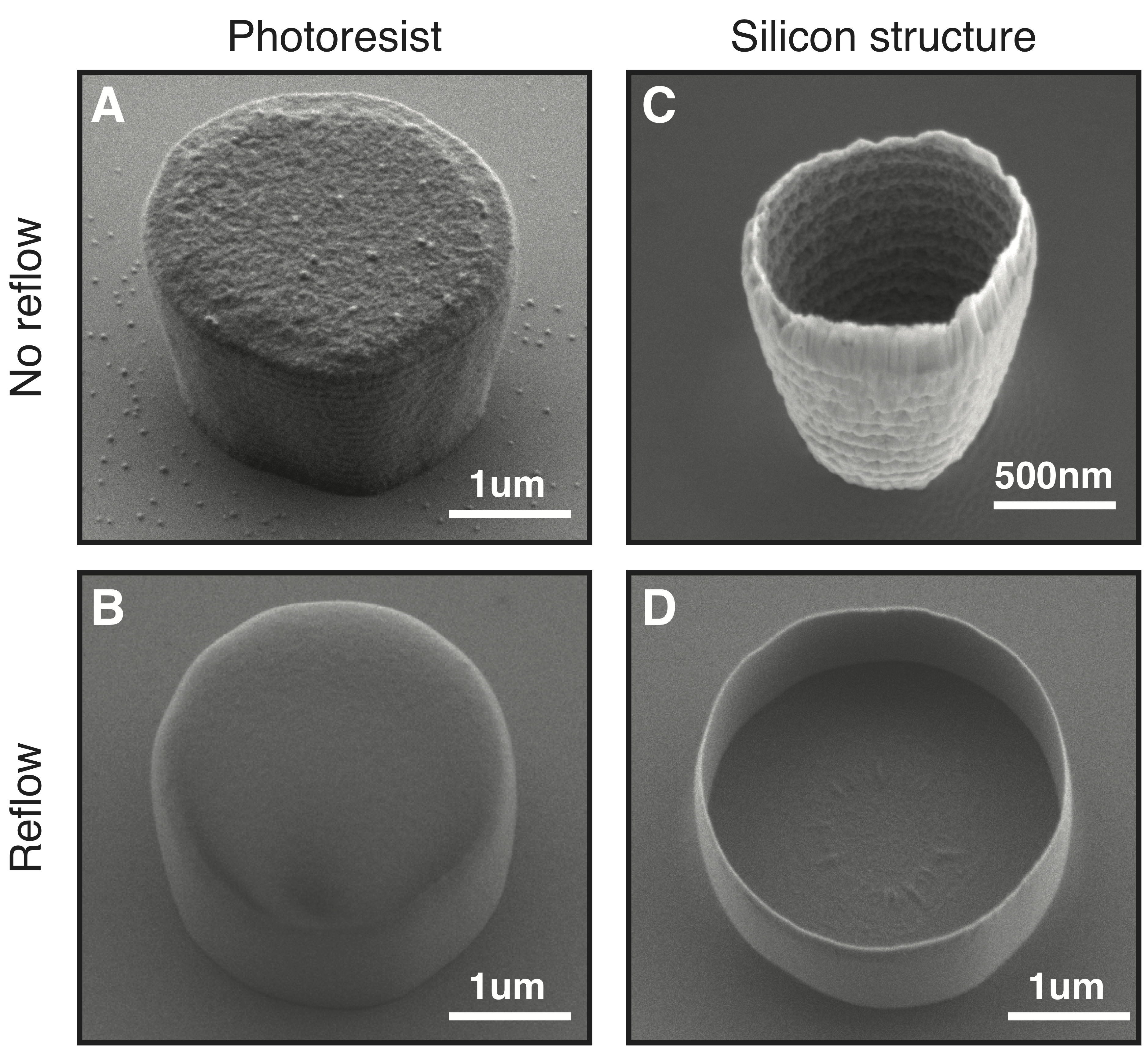

Supplement: Supplementary file 3 — Figure S2 [file 41378_2019_52_MOESM3_ESM.tif]

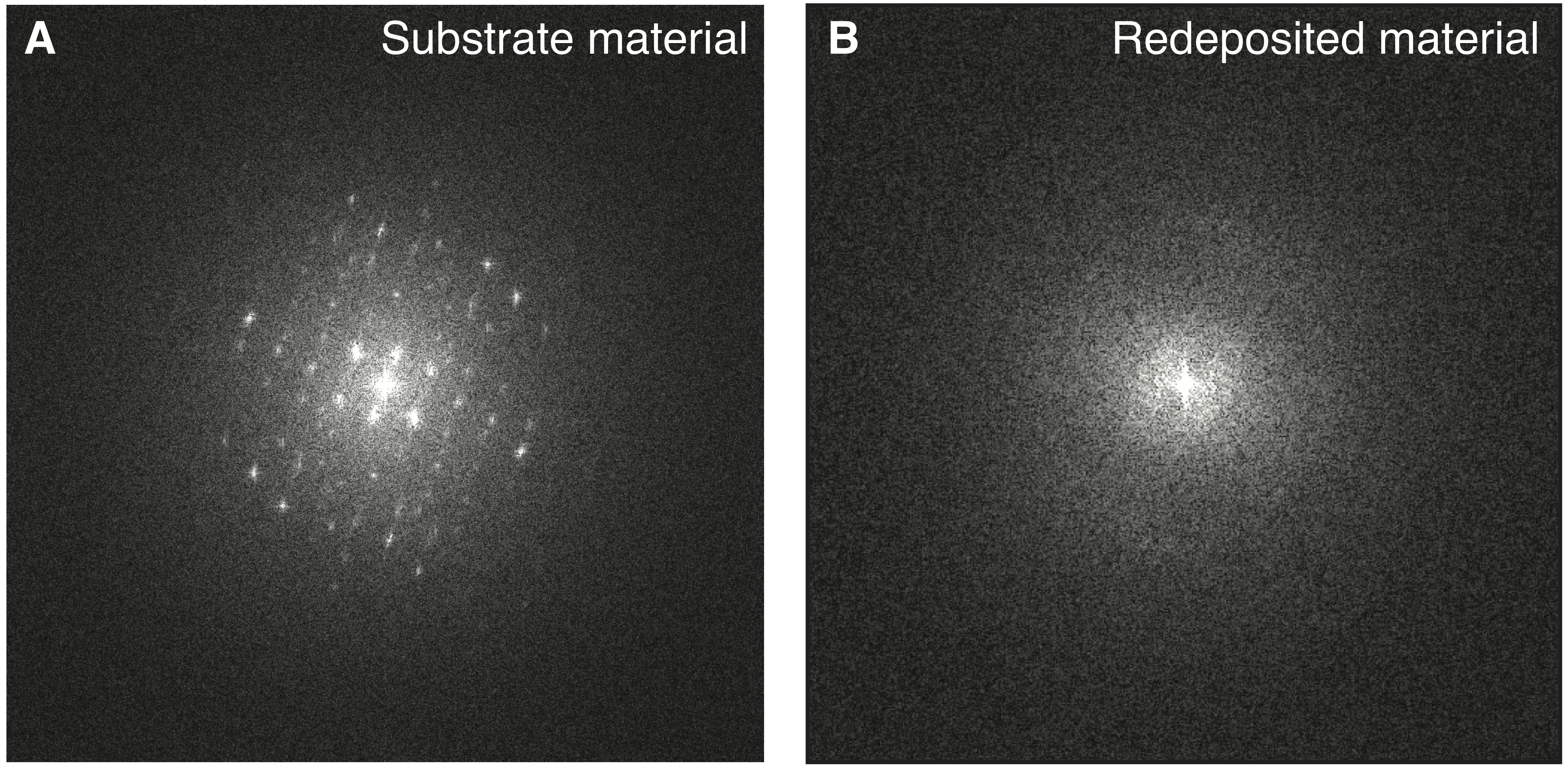

Supplement: Supplementary file 4 — Figure S3 [file 41378_2019_52_MOESM4_ESM.tif]

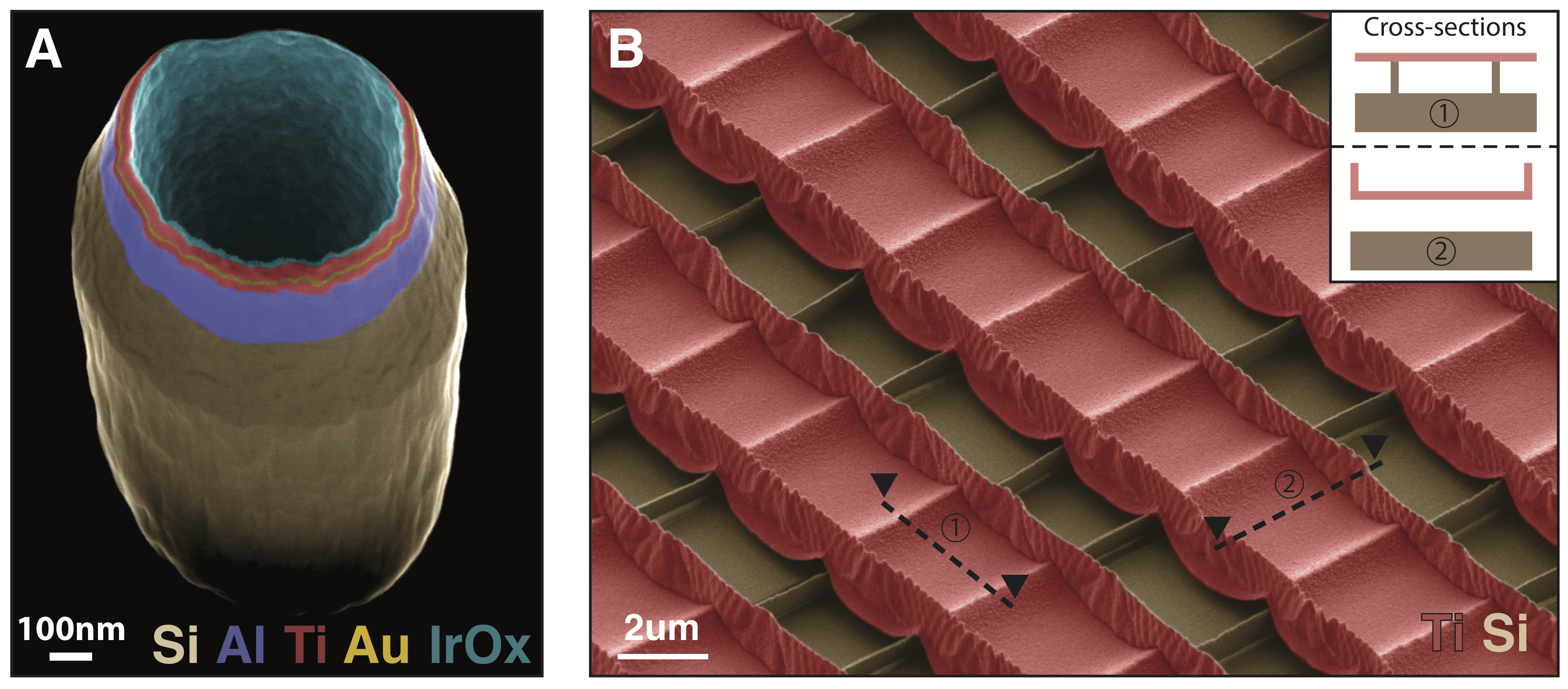

Supplement: Supplementary file 5 — Figure S4 [file 41378_2019_52_MOESM5_ESM.tif]

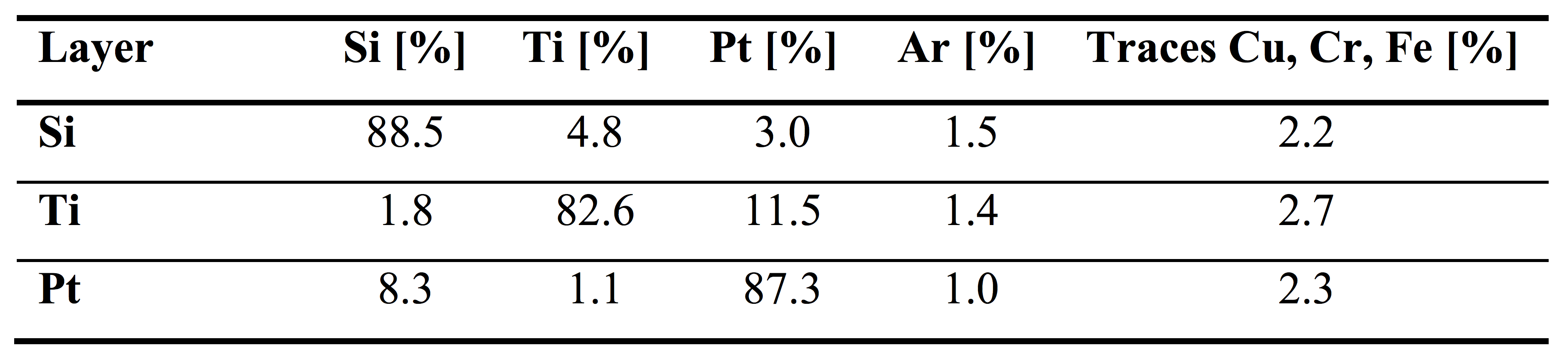

Supplement: Supplementary file 6 — Table S1 [file 41378_2019_52_MOESM6_ESM.tif]
